# Factors influencing the public health emergency preparedness among Generation Z nursing interns in the post-pandemic era: a cross-sectional study

**DOI:** 10.3389/fpubh.2025.1632609

**Published:** 2025-08-29

**Authors:** Chengxu Duan, Guangyi Feng, Xinqi Zhuang, Yenong Zhou, Yitong Jia, Xiaomin Sun, Yin-Ping Zhang

**Affiliations:** ^1^School of Nursing, Xi'an Jiaotong University Health Science Center, Xi'an, Shaanxi, China; ^2^School of Foreign Studies, Xi'an Jiaotong University, Xi'an, Shaanxi, China; ^3^Xi'an No.3 Hospital, The Affiliated Hospital of Northwest University, Xi'an, Shaanxi, China

**Keywords:** public health emergency, preparedness, Generation Z, nursing, post-pandemic era

## Abstract

**Background:**

The frequent occurrence of public health emergencies in the 21st century has posed significant challenges to global healthcare systems. As crucial members of medical teams, Generation Z nursing interns play a pivotal role in emergency response. However, systematic assessments of preparedness encompassing emergency attitude, professional knowledge, operational skills, and comprehensive competencies among nursing interns remain unexplored.

**Objective:**

This study aims to assess the public health emergency preparedness of Generation Z nursing interns in the post-pandemic era and identify key influencing factors, providing insights for optimizing nursing education and clinical training.

**Methods:**

A cross-sectional survey was conducted among 434 Generation Z nursing interns in Zhengzhou, China, from January to February 2025 using convenience sampling. Data were collected using a general information questionnaire, *Public Health Emergency Preparedness Questionnaire for Nursing Interns, Nurse's Digital Competence Self-assessment Scale*, and *Professional Identity Scale for Nursing Students*. Data were analyzed by descriptive statistics, univariate analysis, Pearson's correlation, and multiple stepwise regression.

**Results:**

The total score of public health emergency preparedness of 434 nursing interns was 95.79 ± 11.03, with notable deficiencies in professional knowledge and comprehensive competencies. Multiple stepwise regression analysis revealed that digital competence, professional identity, attitude toward the nursing major, and level of internship hospital were the main influencing factors of emergency preparedness (all *p* < 0.05).

**Conclusion:**

The findings indicate that while Generation Z nursing interns demonstrate a high proficiency in emergency operations and a positive attitude toward emergency response, deficiencies persist in specialized emergency knowledge and psychological intervention capabilities. It is recommended that nursing schools and internship hospitals implement integrated emergency training programs, complemented by enhanced digital competency and professional identity cultivation, to holistically bolster preparedness for future public health emergencies.

## 1 Introduction

A Public Health Emergency is defined as a sudden and unexpected incident that poses significant risks to the health of a population. Such events encompass major infectious disease outbreaks, clusters of unexplained illnesses, severe foodborne or occupational poisoning incidents, and other events that critically endanger population health (1). The frequent occurrence of public health emergencies has posed significant challenges to medical systems globally (2). As crucial members of clinical healthcare teams, nurses play a pivotal role in emergency response and recovery efforts during crises (3). Nursing interns serving as clinical personnel undertake high-intensity, high-risk care responsibilities during such emergencies (4). Throughout the COVID-19 pandemic, several studies (5–9) have documented that the substantial contributions of nursing interns across multilevel healthcare systems, including hospital wards, public health agencies, and community clinics, where they executed tasks spanning patient care, epidemic management, and other emergency response procedures. As China's future frontline clinical nursing workforce, nursing interns constitute both a vital reserve of healthcare talent and an essential emergency response contingent, substantially having a positive impact on the overall effectiveness of healthcare systems in responding to public health emergencies through their contributions (10). Despite their active involvement, the preparedness of nursing interns in addressing public health emergencies remains a concern. Several studies (5, 7, 11, 12) have indicated that nursing interns frequently encounter critical challenges due to their insufficient acquisition of professional knowledge, limited practical experience in crisis response, inadequate psychological resilience, and other competency gaps during public health emergencies, all of which warrant focused attention.

Nursing interns' public health emergency preparedness refers to their readiness level in emergency attitude, professional knowledge, emergency operational skills, and comprehensive competence to competently fulfill emergency healthcare roles during outbreak scenarios (13). Generation Z (born 1995–2009) nursing interns, as the first generation born entirely within the digital age, represent a unique cohort shaped by accelerated digital technology advancement and the transformative impact of COVID-19 pandemic (14). In the post-pandemic era, clinical internships function not only as a pivotal transitional stage for nursing students to develop their professional role as next generation practitioners (15), but also as an integrative platform for contextualizing theoretical knowledge, honing clinical skills, and accumulating emergency expertise, all of which are critical to comprehensively strengthening Generation Z nursing interns' preparedness for public health emergencies. While current studies (16–18) predominantly concentrate on nurses' competencies and improvement strategies in public health emergencies, the emergency preparedness of nursing interns during such crises remains underexplored, particularly research combining digital competence with professional identity. Digital competence enables nursing interns to effectively integrate technologies into clinical practice, thereby enhancing work efficiency and care service quality (19–21) while strengthening public health emergency response capabilities. Furthermore, the complexity of public health emergencies demands not only technical competence from nursing interns but also strong professional commitment. Professional identity, as a paramount intrinsic motivator, may systematically enhance their emergency preparedness through pathways including strengthening accountability, enhancing learning motivation, and bolstering resilience (22–24). Emergency preparedness constitute a foundational element in ensuring effective medical rescue and nursing care delivery during public health emergencies. Neglecting the emergency preparedness of Generation Z nursing interns, who represent an important workforce contingent in modern healthcare systems, may critically compromise future emergency response effectiveness, hinder the formation of specialized nursing rapid response units, and exacerbate preexisting deficiencies in medical systems during pivotal stages of public health emergency (13).

Therefore, this study aims to investigate the emergency preparedness of Generation Z nursing interns in the post-pandemic era through a cross-sectional survey, with the objectives of assessing the level of current preparedness for public health emergencies and identifying critical factors influencing their preparedness. The findings of this study will enable nursing schools and internship hospitals to operationalize precision educational strategies in the post-pandemic era, therefore systematically elevating Generation Z nursing interns' emergency preparedness, ultimately fortifying the adaptive and resilience capacity of healthcare systems when confronted with future public health emergencies.

## 2 Methods

### 2.1 Study participants

A convenience sampling method was used to select qualified nursing interns in Zhengzhou, China from January to February 2025. Inclusion criteria were as follows: (1) nursing students engaging in clinical internship; (2) nursing students born between January 1, 1995, and December 31, 2009; (3) voluntary participation in this study. Exclusion criteria were as follows: (1) clinically diagnosed psychological disorders; (2) being exposed to major life events within the preceding 3 months (e.g., severe illness, family adversity, or bereavement); (3) inability to comprehend survey items or demonstration of patterned responses. This study adhered to the Strengthening the Reporting of Observational Studies in Epidemiology (STROBE) guidelines.

### 2.2 Sample size

The G^*^Power 3.1.9 program was used to calculate the sample size. The minimum sample size required for conducting multiple linear regression analysis was calculated to be 301, with the following conditions: *F* tests - Linear multiple regression: fixed model, *R*^2^ deviation from zero, Effect size *f*^2^ = 0.15, significance level at α = 0.05, number of predictors = 80, and a power of 0.80. Considering a 20% non-response rate, a total of 450 questionnaires were distributed, and 434 valid questionnaires were recovered, with a validity rate of 96.44%.

### 2.3 Variables and instruments

#### 2.3.1 General characteristics

The self-developed questionnaire included questions concerning participants' age, gender, education level, residence, experience of being student leaders, parental healthcare occupation, motivation for choosing nursing major, attitude toward nursing major, level of internship hospital, participation in public health emergency rescue or volunteering, and participation in public health emergency training or simulation drills.

#### 2.3.2 Public Health Emergency Preparedness Questionnaire for Nursing Interns

The *Public Health Emergency Preparedness Questionnaire for Nursing Interns (PHEPQNI)* was developed by Xu (13). The scale contains four dimensions and 25 items, which are emergency attitude (three items), professional knowledge (eight items), emergency operational skills (six items), and comprehensive competence (eight items). Responses are rated on a 5-point Likert scale ranging from 1 (strongly disagree) to 5 (strongly agree), with higher composite scores indicating greater preparedness levels. The total Cronbach's α was 0.923, and the Cronbach's α of the dimensions ranged from 0.784 to 0.908 in this study.

#### 2.3.3 Nurse's Digital Competence Self-Assessment Scale

The *Nurse's Digital Competence Self-assessment Scale (NDCSS)* was developed by Qin et al. (25). The scale contains three dimensions and 27 items, which are digital information retrieval and evaluation (five items), digital methodology application (14 items), digital security and responsibility (eight items). Responses are rated on a 5-point Likert scale ranging from 1 (strongly disagree) to 5 (strongly agree), with higher composite scores indicating stronger digital competency. The total Cronbach's α was 0.982, and the Cronbach's α of the dimensions ranged from 0.909 to 0.969 in this study.

#### 2.3.4 Professional Identity Scale for Nursing Students

The *Professional Identity Scale for Nursing Students (PISNS)* was developed by Hao et al. (26). It is a psychometrically instrument widely used in China to evaluate the level of nursing students' professional identity. The scale contains five dimensions and 17 items, which are professional self-image (six items), benefit of retention and risk of turnover (four items), social comparison and self-reflection (three items), independence of career choice (two items), and social modeling (two items), with item 12 reverse-scored. The original study demonstrated that the total Cronbach's α is 0.830 and subscales range from 0.462 to 0.831. Responses are rated on a 5-point Likert scale ranging from 1 (strongly disagree) to 5 (strongly agree), with higher composite scores indicating stronger professional identity. The total Cronbach's α was 0.969, and the Cronbach's α of the dimensions ranged from 0.478 to 0.962 in this study.

### 2.4 Data collection

With the approval of nursing education departments across multiple hospitals in Zhengzhou, coordinated recruitment of nursing interns was conducted for centralized data collection. The participating hospitals were selected based on the following criteria: (1) major clinical training bases at different levels within Zhengzhou (including Grade-A tertiary, Grade-B tertiary, and Grade-A secondary hospitals); (2) the presence of a substantial cohort of Generation Z nursing interns actively undergoing clinical training within the hospital; (3) the hospital administration expressed willingness to provide support and research facilitation during the study period. Across all participating hospitals, research recruitment information were disseminated by nursing education departments during dedicated educational assemblies where nursing interns were centrally convened for clinical training. Professionally trained researchers explicitly explained the survey objectives and significance to participating nursing interns. With informed consent, Generation Z nursing interns voluntarily completed anonymous questionnaires independently. All questionnaires were collected immediately upon completion, and researchers conducted on-spot quality checks to ensure data authenticity and reliability.

### 2.5 Statistical analysis

The collected data were entered into Excel for validation and screening to ensure accuracy. Qualified datasets were imported into SPSS Statistics 27.0 software program for analysis. For large-sample data, when the absolute values of kurtosis index are < 10 and skew index are < 3 after statistical testing, the data are considered approximately normally distributed (27). Descriptive statistics were expressed as means with standard deviations for continuous variables. Intergroup differences in demographic characteristics were assessed using *t*-tests and analysis of variance (ANOVA). Pearson's correlation analysis was conducted to examine the associations among public health emergency preparedness of nursing interns, digital competency, professional identity, clinical pedagogical atmosphere, and proactive personality traits. A multiple linear regression model was constructed with the total score of public health emergency preparedness among nursing interns as the dependent variable. Statistically significant variables identified through univariate and correlation analyses were included as predictors, followed by stepwise regression procedures, and a value < 0.05 indicated statistical significance.

## 3 Results

### 3.1 Demographic characteristics

A total of 434 valid questionnaires were collected. Participants' ages ranged from 18 to 23 years, consistent with the Generation Z cohort (born 1995–2009) specified in our inclusion criteria, with an average age of 21.81 ± 1.33 years. The majority of the participants were female (83.64%), from rural areas (56.45%), and their parents without medical professional backgrounds (94.47%). Regarding educational background and internship experiences, a majority were undergraduate (50.46%), without a student leader experience (68.66%), undergoing internships within Grade-A tertiary hospital (84.56%). In terms of emergency experiences, more than half of the participants had not participated in emergency rescue or volunteer work during public health emergency (56.45%), while nearly three fifths of the participants (58.99%) had received training or participated in simulation drills for such events. More detailed demographic characteristics of the 434 participants are presented in Table 1.

**Table 1:** Univariate analysis of PHEPQNI for generation Z nursing interns (*n* = 434).

**Variables**	**Category**	**Number of cases (composition ratio) [*n* (%)]**	**Score ($\bar{x}$ ±*s*)**	***t*/*F***	***p*-Value**
Age	18	27 (6.22)	93.44 ± 11.37	1.932	0.088
	19	46 (10.60)	97.09 ± 12.81		
	20	96 (22.12)	94.96 ± 10.71		
	21	117 (26.96)	97.75 ± 10.14		
	22	111 (25.58)	96.71 ± 11.79		
	23	37 (8.53)	92.51 ± 10.80		
Gender	Male	71 (16.36)	95.31 ± 12.46	−0.636	0.525
	Female	363 (83.64)	96.23 ± 10.94		
Education level	Technical secondary school	54 (12.44)	92.50 ± 9.89	3.936	0.020^*^
	Junior college	161 (37.10)	97.40 ± 11.03		
	Undergraduate	219 (50.46)	96.00 ± 11.46		
Residence	Urban area	93 (21.43)	96.78 ± 12.10	2.552	0.079
	County city	96 (22.12)	97.97 ± 10.65		
	Rural area	245 (56.45)	95.08 ± 10.97		
Experience of being student leaders	Yes	136 (31.34)	97.45 ± 11.71	1.721	0.086
	No	298 (68.66)	95.46 ± 10.91		
Parental healthcare occupation	Both parents	7 (1.61)	99.29 ± 8.14	0.789	0.455
	One parent	17 (3.92)	93.29 ± 10.10		
	Neither	410 (94.47)	95.84 ± 11.11		
Motivation for choosing nursing major	Personal interest	148 (34.10)	97.93 ± 10.15	4.354	0.013^*^
	Parental/others' suggestion	229 (52.76)	95.64 ± 11.14		
	Major adjustment	57 (13.13)	93.05 ± 13.17		
Attitude toward nursing major	Strongly dislike	6 (1.38)	81.33 ± 14.46	10.953	< 0.001^**^
	Dislike	28 (6.45)	88.39 ± 11.79		
	Neutral	192 (44.24)	94.61 ± 11.28		
	Like	151 (34.79)	98.95 ± 10.07		
	Very much like	57 (13.13)	98.75 ± 9.39		
Level of internship hospital	Grade-A tertiary hospital	367 (84.56)	96.51 ± 11.12	5.153	0.006^*^
	Grade-B tertiary hospital	56 (12.90)	91.84 ± 10.27		
	Grade-A secondary hospital	11 (2.53)	91.91 ± 2.04		
Public health emergency rescue or volunteering	Yes	189 (43.55)	97.84 ± 10.91	2.899	0.004^**^
	No	245 (56.45)	94.73 ± 11.24		
Public health emergency training or simulation drills	Yes	256 (58.99)	98.38 ± 11.23	5.291	< 0.001^**^
	No	178 (41.01)	92.78 ± 10.30		

^*^p < 0.05.

^**^p < 0.01.

### 3.2 Scores of the main variables

**Figure 1:**
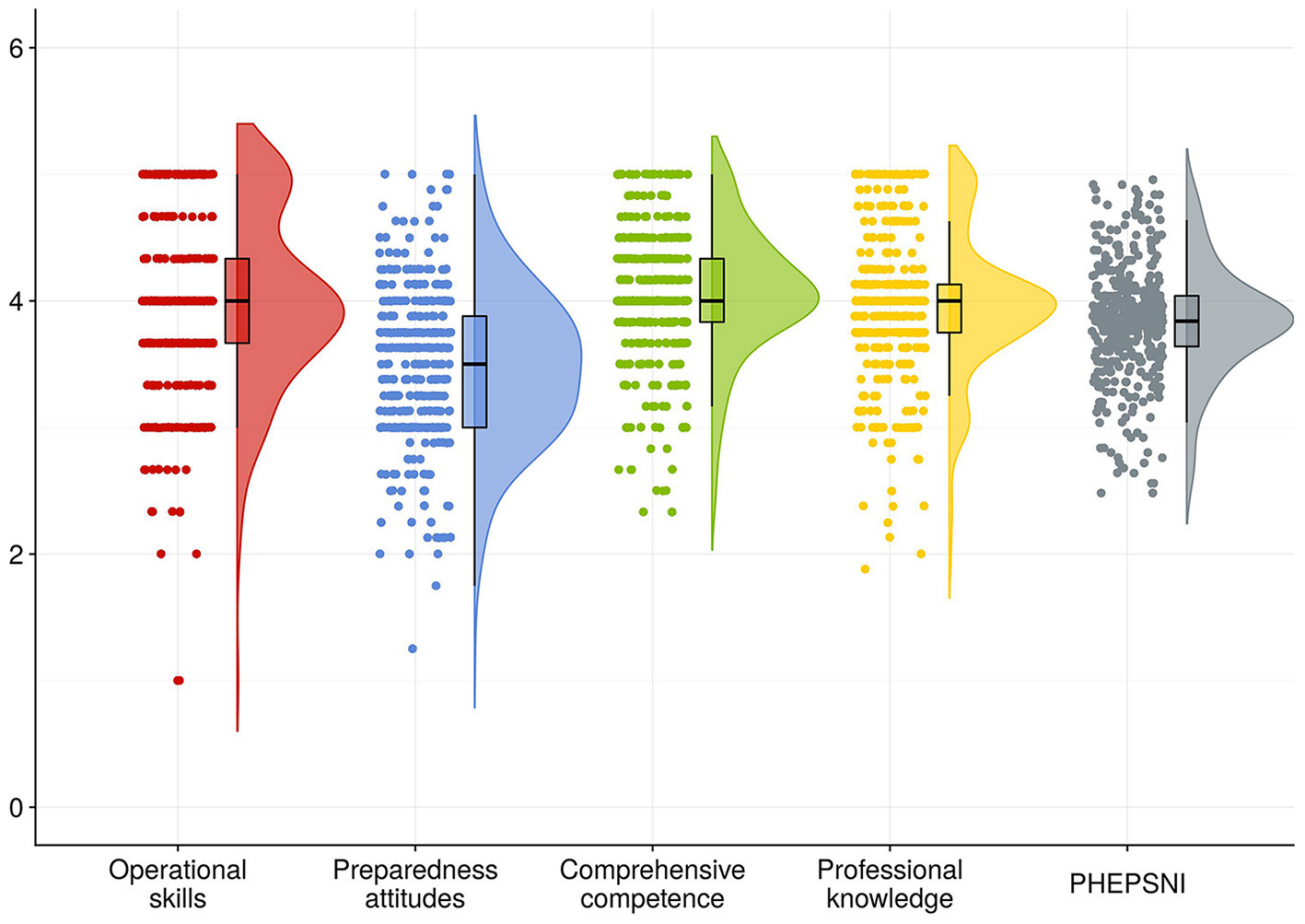
Mean score of items in the dimension of PHEPQNI. The horizontal axis indicated the different dimensions of PHEPQNI, and the vertical axis indicated the average scores of entries in the different dimensions.

**Figure 2:**
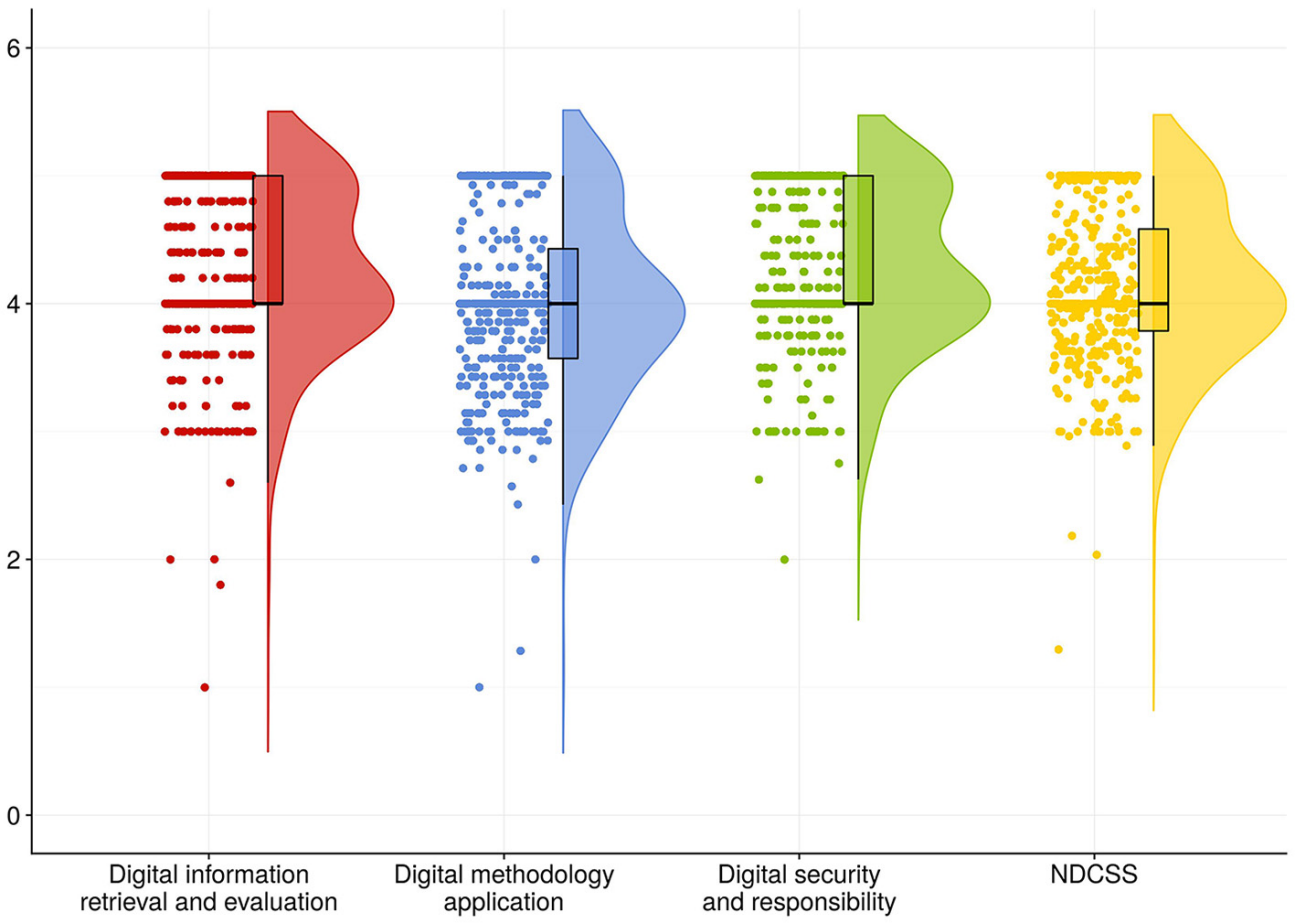
Mean score of items in the dimension of NDCSS. The horizontal axis indicated the different dimensions of NDCSS, and the vertical axis indicated the average scores of entries in the different dimensions.

**Figure 3:**
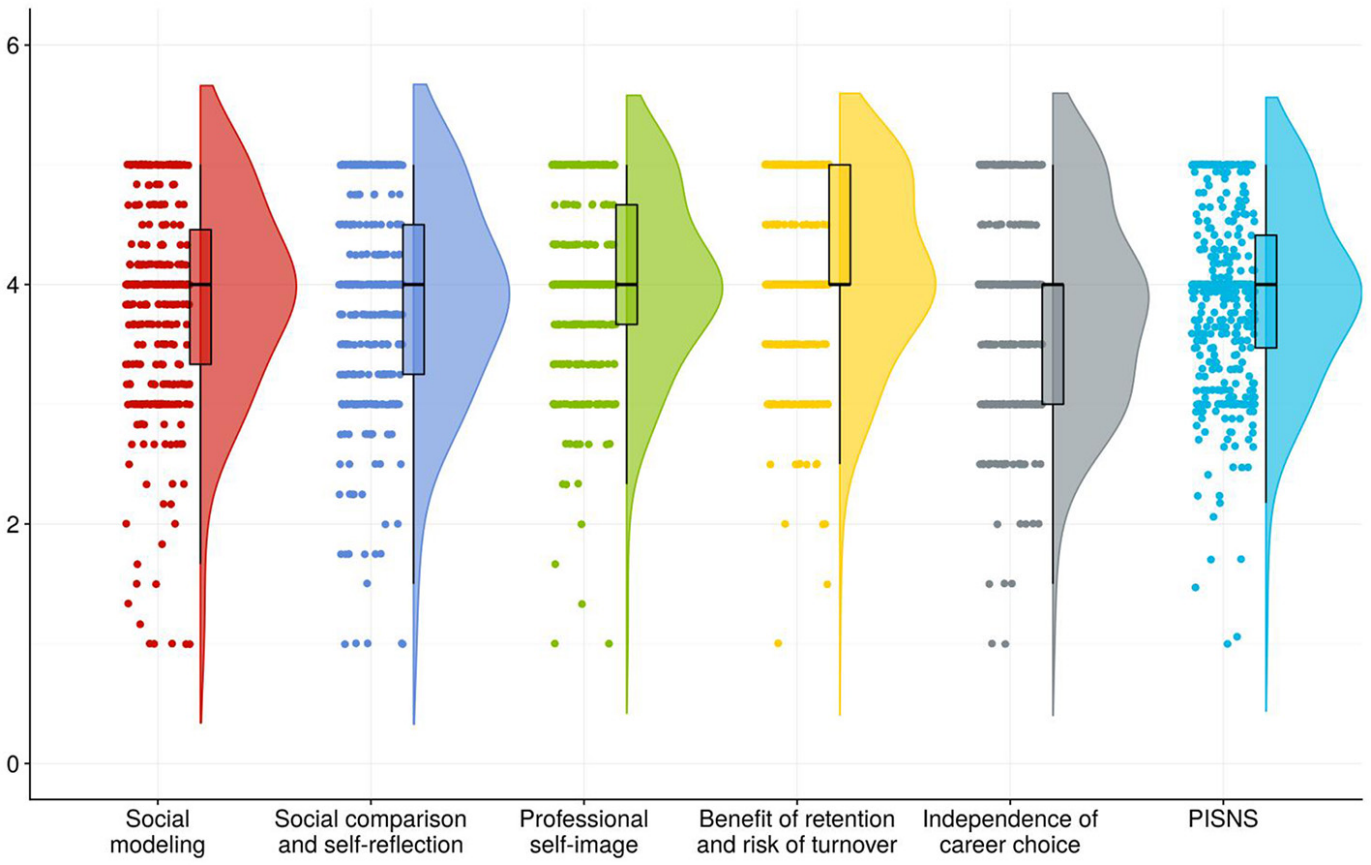
Mean score of items in the dimension of PISNS. The horizontal axis indicated the different dimensions of PISNS, and the vertical axis indicated the average scores of entries in the different dimensions.

In the post-pandemic era, Generation Z nursing interns in this study demonstrated a mean public health emergency preparedness score of 95.79 ± 11.03. The mean score of items in the PHEPQNI, NDCSS and PISNS are detailed in Table 2, Figures 1–3.

**Table 2:** Scores on each dimension of PHEPQNI, NDCSS, and PISNS ($\bar{x}$ ± s, score).

**Scale classification**	**Dimensional ordering**	**Dimensions' name**	**Mean score of items in the dimension**
PHEPQNI		Total	3.84 ± 0.44
	1	Operational skills	4.07 ± 0.49
	2	Preparedness attitudes	4.00 ± 0.70
	3	Comprehensive competence	3.97 ± 0.56
	4	Professional knowledge	3.48 ± 0.58
NDCSS		Total	4.13 ± 0.59
	1	Digital security and responsibility	4.27 ± 0.59
	2	Digital information retrieval and evaluation	4.25 ± 0.63
	3	Digital methodology application	4.01 ± 0.67
PISNS		Total	3.92 ± 0.74
	1	Social modeling	4.12 ± 0.75
	2	Social comparison and self-reflection	4.02 ± 0.72
	3	Professional self-image	3.90 ± 0.82
	4	Benefit of retention and risk of turnover	3.85 ± 0.84
	5	Independence of career choice	3.79 ± 0.75

Within the 25 items of PHEPQNI, the three lowest-scoring items were: “Demonstrating knowledge of radiation-related illnesses and nuclear exposure protective measures” (3.23 ± 0.75), “Understanding clinical manifestations and emergency protocols for biochemical attack-induced pathologies, including symptom recognition and immediate interventions” (3.41 ± 0.79), and “Understanding key provisions outlined in *Regulations on Emergency Response to Public Health Incidents*” (3.41 ± 0.80). For each scale, the three lowest-scoring items ranked in descending order of their mean values were detailed in Table 3.

**Table 3:** Enumeration of items with the lowest scores on the PHEPQNI, NDCSS, and PISNS ($\bar{x}$ ± s, score).

**Scale**	**Content of items**	**Affiliated dimensions**	**Scores**
PHEPQNI	Demonstrating knowledge of radiation-related illnesses and protective measures against nuclear exposure	Professional knowledge	3.23 ± 0.75
	Understanding clinical manifestations and emergency care for biochemical attack-induced pathologies, including symptom recognition and immediate intervention strategies	Professional knowledge	3.41 ± 0.79
	Understanding key contents of Regulations on Emergency Response to Public Health Emergencies	Professional knowledge	3.41 ± 0.80
NDCSS	I can analyze nursing challenges through digital health technologies	Digital methodology application	3.90 ± 0.82
	I can implement digital innovations to transform nursing practice	Digital methodology application	3.94 ± 0.80
	I can enhance clinical decisions through digital health technologies	Digital methodology application	3.94 ± 0.80
PISNS	I derive my professional understanding exclusively from external authorities (e.g., parents, teachers et al.) without critical self-reflection	Independence of career choice	3.51 ± 1.19
	Career transition from nursing would cause me emotional distress	Benefit of retention and risk of turnover	3.67 ± 1.06
	Working as a nurse brings me joy	Professional self-image	3.81 ± 0.92

### 3.3 Influencing factors of Generation Z nursing interns' public health emergency preparedness

#### 3.3.1 Univariate analysis

As shown in Table 1, the univariate analysis revealed significant differences in Generation Z nursing interns' emergency preparedness scores across the following factors: education level, motivation for choosing nursing, attitude toward nursing profession, level of intern hospital, participation in public health emergency rescue or volunteer work and participation in public health emergency training or simulation drills (*p* < 0.05).

#### 3.3.2 Correlations between PHEPQNI, NDCSS, and PISNS

As shown in Table 4, Pearson correlation analysis revealed that the total score of PHEPQNI statistically significant positive correlated with the total score of the NDCSS, digital information retrieval and evaluation, digital methodology application, digital security and responsibility (*r* = 0.431–0.530, *p* < 0.05). The total score of PHEPQNI also positively correlated with the total score of PISNS, professional self-image, benefit of retention and risk of turnover, social comparison and self-reflection, independence of career choice, and social modeling (*r* = 0.289–0.442, *p* < 0.05).

**Table 4:** Correlation between public health emergency preparedness, digital competence, and professional identity.

**Dimensions**	**Preparedness attitudes**	**Professional knowledge**	**Operational skills**	**Comprehensive competence**	**Total score of PHEPQNI**
**NDCSS dimensions**					
Digital information retrieval and evaluation	0.520^**^	0.202^**^	0.403^**^	0.485^**^	0.489^**^
Digital methodology application	0.554^**^	0.290^**^	0.390^**^	0.482^**^	0.528^**^
Digital security and responsibility	0.506^**^	0.124^**^	0.361^**^	0.459^**^	0.431^**^
Total of NDCSS scores	0.574^**^	0.245^**^	0.412^**^	0.511^**^	0.530^**^
**PISNS dimensions**					
Professional self-image	0.513^**^	0.227^**^	0.259^**^	0.284^**^	0.378^**^
Benefit of retention and risk of turnover	0.533^**^	0.247^**^	0.273^**^	0.301^**^	0.400^**^
Social comparison and self-reflection	0.580^**^	0.191^**^	0.374^**^	0.373^**^	0.442^**^
Independence of career choice	0.469^**^	0.156^**^	0.204^**^	0.195^**^	0.289^**^
Social modeling	0.554^**^	0.215^**^	0.321^**^	0.374^**^	0.433^**^
Total of PISNS scores	0.572^**^	0.234^**^	0.305^**^	0.327^**^	0.422^**^

^**^p < 0.01.

#### 3.3.3 Multiple stepwise linear regression analysis

A multiple stepwise linear regression analysis was conducted to examine influencing factors of public health emergency preparedness for Generation Z nursing interns. The emergency preparedness scores of nursing interns was used as the dependent variable and eight variables with statistical significance identified in univariate and correlation analyses were included as independent variables. Variable assignments and dummy variable coding are detailed in Table 5. The results demonstrated that the level of the internship hospital, digital competence of nursing, and professional identity significantly were factors influencing emergency preparedness for public health emergency for nursing interns (*p* < 0.05). More detailed results of multiple stepwise linear regression are presented in Table 6.

**Table 5:** Independent variable assignment and dummy variable setting.

**Considerations**	**Variable name**	**Description of the assignment**
Education level	X1	Set dummy variable
	UndergraduateX11	X11 = 1, X12 = 0, X13 = 0
	Junior collegeX12	X11 = 0, X12 = 1, X13 = 0
	Secondary specialized schoolX13	X11 = 0, X12 = 0, X13 = 0 (Reference)
Motivation for choosing nursing	X2	Set dummy variable
	Personal interestX21	X21 = 1, X22 = 0, X23 = 0
	Parental/others' suggestionX22	X21 = 0, X22 = 1, X23 = 0
	Major adjustmentX23	X21 = 0, X22 = 0, X23 = 0 (Reference)
Attitude toward nursing major	X3	Set dummy variable
	Strongly likeX31	X31 = 1, X32 = 0, X33 = 0, X34 = 0, X35 = 0
	LikeX32	X31 = 0, X32 = 1, X33 = 0, X34 = 0, X35 = 0
	NeutralX33	X31 = 0, X32 = 0, X33 = 1, X34 = 0, X35 = 0
	DislikeX34	X31 = 0, X32 = 0, X33 = 0, X34 = 1, X35 = 0
	Strongly dislikeX35	X31 = 0, X32 = 0, X33 = 0, X34 = 0, X35 = 0 (Reference)
Level of internship hospital	X4	Set dummy variable
	Grade-A tertiary hospitalX41	X41 = 1, X42 = 0, X43 = 0
	Grade-B tertiary hospitalX42	X41 = 0, X42 = 1, X43 = 0
	Grade-A secondary hospitalX43	X41 = 0, X42 = 0, X43 = 0 (Reference)
Public health emergency rescue or volunteer work	X5	No = 0, Yes = 1
Public health emergency training or simulation drills	X6	No = 0, Yes = 1
NDCSS	X7	Input as original value
PISNS	X8	Input as original value
PHEPQNI	Y	Input as original value

**Table 6:** Multiple stepwise linear regression analysis of factors influencing the public health emergency preparedness (*n* = 434).

**Variables**	***B***	**SE**	**β**	***t***	***p-*Value**	**VIF**
Constant	48.176	3.451		14.407	< 0.001	
NDCSS	0.307	0.038	0.446	8.172	< 0.001	1.909
PISNS	0.106	0.049	0.121	2.176	0.030	1.983
Grade-A tertiary hospital (Level of internship hospital)	3.118	1.225	0.102	2.545	0.011	1.031
Like (Attitude toward nursing major)	5.573	1.209	0.241	4.609	< 0.001	1.747
Neutral (Attitude toward nursing major)	3.863	1.186	0.174	3.258	0.001	1.825

SE, standard error; β, standardized regression coefficient; F = 42.308, p < 0.001, R^2^ = 0.331, D-W = 1.884.

The variance inflation factor (VIF) was < 10, so there was no multicollinearity.

## 4 Discussion

### 4.1 The current status of public health emergency preparedness for Generation Z nursing interns

This study revealed that the overall emergency preparedness score among 434 nursing interns was 95.79 ± 11.03. This may be attributed to the self-improvement of nursing interns in competency, the accumulation of practical experience, and the enhancement of nursing disaster education. Firstly, frequent public health emergencies, notably the COVID-19 pandemic and mpox outbreak, have driven nursing students to proactively engage in acquiring emergency preparedness knowledge and refining risk perception capabilities. Participation in volunteer services and pandemic management has systematically accumulated substantial professional response experience among nursing interns (28–30). Secondly, nursing education systems have improved curriculum design by introducing specialized disaster nursing courses that focus on public health emergency response since the outbreak of the COVID-19 pandemic (31, 32). These courses incorporate targeted instructional content that effectively enhances nursing students' crisis response competencies and qualifies them for public health emergency preparedness.

The analysis of dimensions demonstrated that the lowest scores emerged in professional knowledge, particularly in the understanding of chemical, biological, radiological, nuclear (CBRN) protection and healthcare legal and regulatory knowledge. The deficiencies may be attributed to nursing education systems focusing on general public health emergency preparedness while lacking systematic educational frameworks for specialized domains such as CBRN protection and disaster medical response strategies (33, 34). During clinical internship, nursing interns rarely encounter knowledge or experience related to such special public health emergencies, exacerbating in insufficient mastery of relevant professional knowledge. Legal education's abstract nature further diminishes the engagement of learning motivation, exacerbating insufficient mastery of relevant professional knowledge (35). Therefore, it is recommended that nursing colleges establish systematic training curricula emphasizing emergency care for natural disasters, CBRN protection, and nursing-related legal education. Internship hospitals should adopt innovative pedagogical approaches, such as implementing scenario-based simulation drills for CBRN attack or natural disaster casualties and conducting practical assessments to reinforce students' understanding of specialized knowledge. Meanwhile, nursing colleges and internship hospitals should jointly organize legal knowledge seminars and develop interdisciplinary legal courses to strengthen students' legal literacy and improve their comprehension of nursing-related laws and regulations.

This study indicated that the comprehensive competency of Generation Z nursing interns in responding to public health emergencies is relatively inadequate, particularly in psychological assessment and health education capabilities. Several studies (36–38) have showed that inadequately prioritizing learning health education and psychological intervention curricula during academic training resulting in nursing students' superficial mastery of psychological care competencies. During clinical internship, health education responsibilities predominantly fall to clinical preceptors due to the transitional role of nursing interns (37). In addition, aligning with the ICN Core Competencies in Disaster Nursing (39), Generation Z nursing interns should require prioritized development in psychological care competencies. Therefore, nursing schools should enhance psychological assessment training in public health emergency courses, while hospitals should provide structured health education practice to improve nursing interns' comprehensive competency in managing patients' psychological crises.

### 4.2 Analysis of the influencing factors of emergency preparedness for public health emergency for nursing interns

#### 4.2.1 Digital competence

Digital competence refers to nursing professionals' ability to utilize digital technologies with confidence, responsibility, and critical thinking in clinical practice, education, and social engagement (25). This study quantified nursing interns' digital competence at 111.59 ± 16.06, reflecting upper-moderate proficiency that is potentially attributed to their status as digital natives from Generation Z who possess inherent technological adaptability (40, 41). However, a concerning deficiency emerges in Generation Z nursing interns' digital technology application competence, particularly in synthesizing technological applications with clinical practice requirements (42). These underperforming indicators directly mirror the operational demands of digital preparedness required for effective public health emergency response systems. Several studies (20, 43) have demonstrated that inadequate digital competencies among nursing professionals exacerbate technology-related anxiety, impede adaptation to digital healthcare transformations, and ultimately undermine the effectiveness of emergency response. The potential reasons for the limited digital application competence among Generation Z nursing interns may stem from the absence of a systematic theoretical framework in cultivating higher-order digital literacy and skills during academic training. Current educational interventions predominantly emphasize fragmented aspects such as digital content analysis and rudimentary operational training for clinical devices, which fail to bridge the competency gap required for a complex clinical environment and emergency digital utilization scenarios (44). During clinical internship, nursing interns are predominantly assigned primary care tasks, limiting their opportunities to engage with healthcare digital systems and autonomously apply clinical technologies in complex cases (45). Notably, in the regression model, digital competence exhibited the highest standardized regression coefficient (β = 0.412, *p* < 0.001), indicating its dominant effect size relative to other predictors, which implies that educational interventions focused on digital competence may yield greater improvements in public health emergency preparedness. To bridge this competency gap in the post-pandemic era, it is necessary to integrate relevant training modules in public health emergency courses, which focus on clinical applications mediated by digital technology rather than technical familiarity. Concurrently, internship hospitals should implement emergency drills requiring nursing interns to utilize digital technology to execute emergency response, thereby enhancing emergency preparedness through contextualized skill application.

#### 4.2.2 Professional identity

This study showed that Generation Z nursing interns exhibit a high level of professional identity, which is an influencing factor of public health emergency preparedness. The enhanced societal recognition of nursing science in COVID-19 pandemic, as well as optimized post-pandemic internship programs, have reinforced Generation Z nursing interns' professional identity through positive valuation (46, 47). Zhang et al. (23) label professional identity as a critical determinant of nursing students' emergency attitudes, suggesting that nursing students with higher professional identity exhibit more proactive responses and positive attitude during crises. Positive emergency attitudes drive nursing interns to improve their emergency preparedness while boosting confidence and willingness to participate in rescue efforts (48). The finding of this study implies that nursing education systems in the post-pandemic era require integrated strategies to strengthen professional identity formation. The regression model demonstrated a relatively high effect size for professional identity (β = 0.121, *p* = 0.030), suggesting that interventions targeting professional identity enhancement among nursing students may serve as a significant pathway to elevate their preparedness for public health emergencies. Nursing schools should establish structured psychological support programs and career exploration workshops during academic training, while hospitals must implement clinical internship environment optimization measures and reduce work pressure. Through all these interventions, Generation Z nursing interns systematically can internalize the profession's core principles and societal significance, elevate professional identity and ultimately enhance emergency preparedness for public health.

#### 4.2.3 Level of internship hospital

This study indicated that Generation Z nursing interns in Grade-A tertiary hospitals exhibited superior preparedness scores for public health emergencies. The reasons may be as follows: designated as China's highest-tier medical institutions, Grade-A tertiary hospitals possess abundant healthcare resources, operate with comprehensive departmental structures including specialized units such as infectious disease departments, fever clinics, and emergency departments (49, 50). During the 8–10-month clinical rotation cycle, Generation Z nursing interns at Grade-A tertiary hospitals undergo training in specialized units. This training program enables their systematic mastery of core competencies, including critical care and infection prevention. Additionally, Grade-A tertiary hospitals established a comprehensive emergency management framework spanning prevention, surveillance, crisis resolution, and staff training (51). Generation Z nursing interns are integrated into public health emergency responding and training systems within these hospitals, and their public health emergency preparedness are cultivated through simulated and actual emergency scenarios (49). However, lower preparedness levels among Generation Z nursing interns in non-tertiary hospitals underscore systemic disparities. Compared with Grade-A tertiary hospitals, these hospitals exhibit significant gaps in clinical training quality, emergency resource reserves, and response mechanisms (51). Given the universal responsibility of multi-level healthcare institutions in public health emergency response, regional collaborative frameworks enabling cross-hospital emergency training resource sharing should take prioritization in the post-pandemic era. Therefore, standardized emergency courses should be delivered to grassroots hospitals through a remote teaching platform led by Grade-A tertiary hospitals, and the assessment of emergency preparedness for nursing interns in grassroots hospitals should be incorporated into teaching indicators. Through the above measures, the emergency preparedness level of Generation Z nursing interns in internship hospitals at different levels can be systematically improved.

#### 4.2.4 Attitude toward nursing major

This study indicated that Generation Z nursing interns with a more proactive attitude toward nursing major exhibited significantly higher emergency preparedness scores for public health emergencies compared with those with a lower passion for the nursing major. Attitude toward nursing major refers to nursing students' cognitive understanding and emotional responses toward the nursing profession, which collectively shape an enduring psychological disposition that directly governs their behavioral patterns (52). Generation Z nursing interns demonstrating a positive attitude manifested intrinsic motivation that stimulated profound interest in acquiring emergency response knowledge (53, 54). This self-driven learning propensity facilitated sustained engagement in mastering critical skills, thereby establishing substantive cognitive reserves through autonomous knowledge accumulation. Furthermore, Generation Z nursing interns with positive attitude toward nursing major displayed heightened endorsement to nursing's vocational values (55). The permeation of vocational values arouses Generation Z nursing interns' strong sense of professional calling, leading them to actively assume occupational responsibilities and voluntarily strengthen learning in public health emergency response competency (56). Therefore, nursing schools and internship hospitals should strengthen professional advocacy and emotional inculcation to enhance Generation Z nursing interns' comprehension of nursing's intrinsic value and foster greater professional enthusiasm, thereby cultivating a positive attitude toward nursing major and elevating Generation Z nursing interns' public health emergency preparedness.

## 5 Conclusion

This study reveals that Generation Z nursing interns in the post-pandemic era demonstrate an upper-moderate level of public health emergency preparedness, with digital competence, professional identity, the level of internship hospital and attitude toward nursing major emerging as critical determinants. While their emergency attitudes reflect strong professional responsibility, gaps exist in knowledge of specialized fields, psychological crisis intervention skills, and legal regulatory awareness. For Generation Z nursing interns exhibiting strong technological adaptability with limited crisis exposure, nursing schools and internship hospitals should prioritize the training in professionalism, digital competencies, and stress resilience. Amidst the ongoing evolution of global health systems in the post-pandemic era, targeted cultivation of public health emergency preparedness among Generation Z nursing interns is fundamental to establishing sustainable emergency response systems.

## 6 Limitation

This study has several limitations that warrant consideration. First, the cross-sectional design inherently restricts causal inference between identified factors and public health emergency preparedness. Second, as Zhengzhou is a provincial capital with relatively concentrated medical resources within Henan Province, the data from nursing interns in this city cannot adequately capture regional disparities, thus limiting the generalizability of our findings to other healthcare systems and regions with scarce healthcare resources. Third, self-reported data may introduce social desirability bias. Within China's healthcare culture that emphasizes altruistic dedication, this could lead participants to overestimate their emergency response capabilities. Fourth, due to the limitations in the study scope and sample size, other potential influencing factors could not be examined, suggesting that future research should expand the scope by investigating additional multidimensional factors for in-depth analysis. Moreover, it should be noted that the instrument for emergency preparedness used in this study was primarily developed based on the Chinese emergency management framework. Consequently, its applicability may be limited within healthcare systems of other cultural contexts. Furthermore, the relatively low reliability of PISNS subscales may lead to diminished effect sizes, potentially resulting in underestimation of the association between professional autonomy and emergency preparedness. Future research should employ longitudinal designs with multi-regional sampling to validate these findings and conduct intervention studies, which could contribute to improving the public health emergency preparedness of the whole Generation Z nursing interns.

## Data Availability

The raw data supporting the conclusions of this article will be made available by the authors, without undue reservation.
